# Mental Health and Burnout Syndrome Among Postgraduate Students in Medical and Multidisciplinary Residencies During the COVID-19 Pandemic in Brazil: Protocol for a Prospective Cohort Study

**DOI:** 10.2196/24298

**Published:** 2021-01-19

**Authors:** Rebeca Da Nóbrega Lucena Pinho, Thais Ferreira Costa, Nayane Miranda Silva, Adriana F Barros-Areal, André De Mattos Salles, Andrea Pedrosa Oliveira, Carlos Rassi, Caroline Elizabeth Brero Valero, Ciro Martins Gomes, Dayde Mendonça-Silva, Fernando Oliveira, Isadora Jochims, Ivan Ranulfo, Juliana De Brito Seixas Neves, Lucas Oliveira, Maria Nogueira Dantas, Marta Rosal, Mayra Soares, Patrícia Kurizky, Viviane Uliana Peterle, Yasmin Furtado Faro, Ana Paula Gomides, Licia da Mota, Cleandro Albuquerque, Cezar Kozak Simaan, Veronica M Amado

**Affiliations:** 1 Programa de Pós-Graduação em Ciências Médicas FM-UnB Brasília Brazil; 2 Hospital Universitário de Brasília - HUB-UnB Brasília Brazil; 3 Secretaria de Estado de Saúde do Distrito Federal - SES DF Brasília Brazil; 4 Universidade de Brasília - UnB Brasília Brazil; 5 Hospital Sírio-Libanês Brasília Brazil; 6 Empresa Brasileira de Serviços Hospitalares - EBSERH Brasília Brazil; 7 Núcleo de Medicina Tropical Universidade de Brasília - UnB Brasília Brazil; 8 Faculdade de Medicina Universidade de Brasília FM-UnB. Brasília Brazil; 9 Universidade Federal do Piauí - UFPI Teresina Brazil; 10 Escola Superior de Ciências da Saúde / ESCS Brasília Brazil; 11 Centro Universitário de Brasília - Uniceub Brasília Brazil

**Keywords:** burnout syndrome, medical residency, multidisciplinary residency, COVID-19, mental health, burnout, stress, anxiety, prospective, cohort, health care professional, medical student

## Abstract

**Background:**

The COVID-19 pandemic has led to high levels of physical, psychological, and social stress among health care professionals, including postgraduate students in medical and multidisciplinary residencies. This stress is associated with the intense fear of occupational exposure to SARS-CoV-2, the virus known to cause COVID-19. These professionals are at risk of developing physical and mental illnesses not only due to the infection but also due to prolonged exposure to multidimensional stress and continued work overload.

**Objective:**

This study aims to evaluate the prevalence of symptoms suggestive of mental disorders and burnout syndrome and determine the risk factors for burnout among postgraduate students in medical and multidisciplinary residencies in Brazil during the COVID-19 pandemic.

**Methods:**

For this prospective cohort study with parallel groups, participants were recruited between July and September 2020 to achieve a sample size of at least 1144 participants. Research instruments such as Depression, Anxiety, and Stress Scale; Patient Health Questionnaire; Brief Resilient Coping Scale; and Oldenburg Burnout Inventory will be used to collect data. Data will be collected in 2 waves: the first wave will include data related to sample characterization and psychosocial evaluation, and the second wave will be launched 12 weeks later and will include an evaluation of the incidence of burnout as well as correlations with the potential predictive factors collected in the first wave. Additionally, we will collect data regarding participants’ withdrawal from work.

**Results:**

The recruitment took place from July 29 to September 5, 2020. Data analyses for this phase is already in progress. The second phase of the study is also in progress. The final data collection began on December 1, 2020, and it will be completed by December 31, 2020.

**Conclusions:**

We believe the findings of this study will help evaluate the impact of the COVID-19 pandemic on the mental health conditions of health professionals in Brazil as well as contribute to the planning and implementation of appropriate measures that can alleviate these mental health challenges.

**International Registered Report Identifier (IRRID):**

DERR1-10.2196/24298

## Introduction

The first COVID-19 outbreak occurred in Wuhan, China, at the end of 2019, and it rapidly spread across the world. On January 30, 2020, the World Health Organization declared that the outbreak constituted a public health emergency of international importance and characterized COVID-19 as a pandemic on March 11, 2020 [1].

During this pandemic, health care professionals, including postgraduate students in medical residency and multidisciplinary programs, have been directly involved in disease management, and consequently, they are exposed to an increased risk of infection due to direct contact with infected patients [2]. Additionally, most of these professionals are likely to develop psychological distress and other mental health–related symptoms, which may be attributed to the lack of security in the face of the unprecedented scenario, increase in the number of confirmed COVID-19 cases, work overload, shortage of diagnostic tests and personal protective equipment (PPE), and the lack of specific drugs for treatment, among other factors [3].

Mental disorders among health care professionals have been the focus of many scientific studies in recent years. A high prevalence of mental health conditions has been reported among the professionals, with a wide spectrum of manifestations correlated to the intense emotional demands and adverse working conditions experienced by them. Physicians and nursing professionals, especially nurses [4], are particularly more susceptible to the development of these problems, in addition to the high levels of work-related stress [5].

In this context, the burnout syndrome stands out. It is defined as a state of physical and mental exhaustion resulting from work activities or care provision, reflected through emotional change and irritability. Burnout is characterized as a psychological syndrome resulting from a continuous response to chronic stressors and interpersonal factors at work. Consequently, psychiatric problems may develop, featuring as emotional exhaustion, depersonalization, and reduced personal achievement [6].

Medical and multidisciplinary residency programs have high workloads and demands; these programs call for many hours of dedication from postgraduate students to fulfill the established requirements. In addition, there can be a significant degree of burnout, which can interfere with the students’ decision-making. In general, burnout syndrome is associated with a number of unfavorable consequences such as depression, risk of medical errors, and patient safety risks [5]. Moreover, an increased workload during this critical period of residency has been found to be associated with a decline in the mental health of the residents [6]; these findings have important implications especially in the ongoing pandemic situation.

In recent years, several studies related to burnout syndrome in health care professionals have been published; however, data published in the scientific literature referring to residents are limited [7]. Studies addressing mental health and burnout syndrome among health care professionals in Brazil and worldwide are quite restricted with regard to the number of residents (ie, medical residents and multidisciplinary residents). Additionally, no studies on these disorders have been performed during the COVID-19 pandemic. Therefore, this study aims to evaluate the prevalence of symptoms suggestive of mental disorders and burnout syndrome as well as determine the risk factors for burnout among postgraduate students in medical and multidisciplinary residencies in Brazil during the COVID-19 pandemic.

## Methods

### Study Design and Data Collection

This is a prospective cohort study comprising 2 parallel groups. Baseline evaluation will be performed at the time of recruitment of study participants and will serve as cross-sectional data to estimate the prevalence of symptoms indicative of mental disorders and professional burnout. A longitudinal follow-up will also be performed to enable estimation of the incidence and identification of predictive factors of burnout among the study participants.

**Figure 1:**
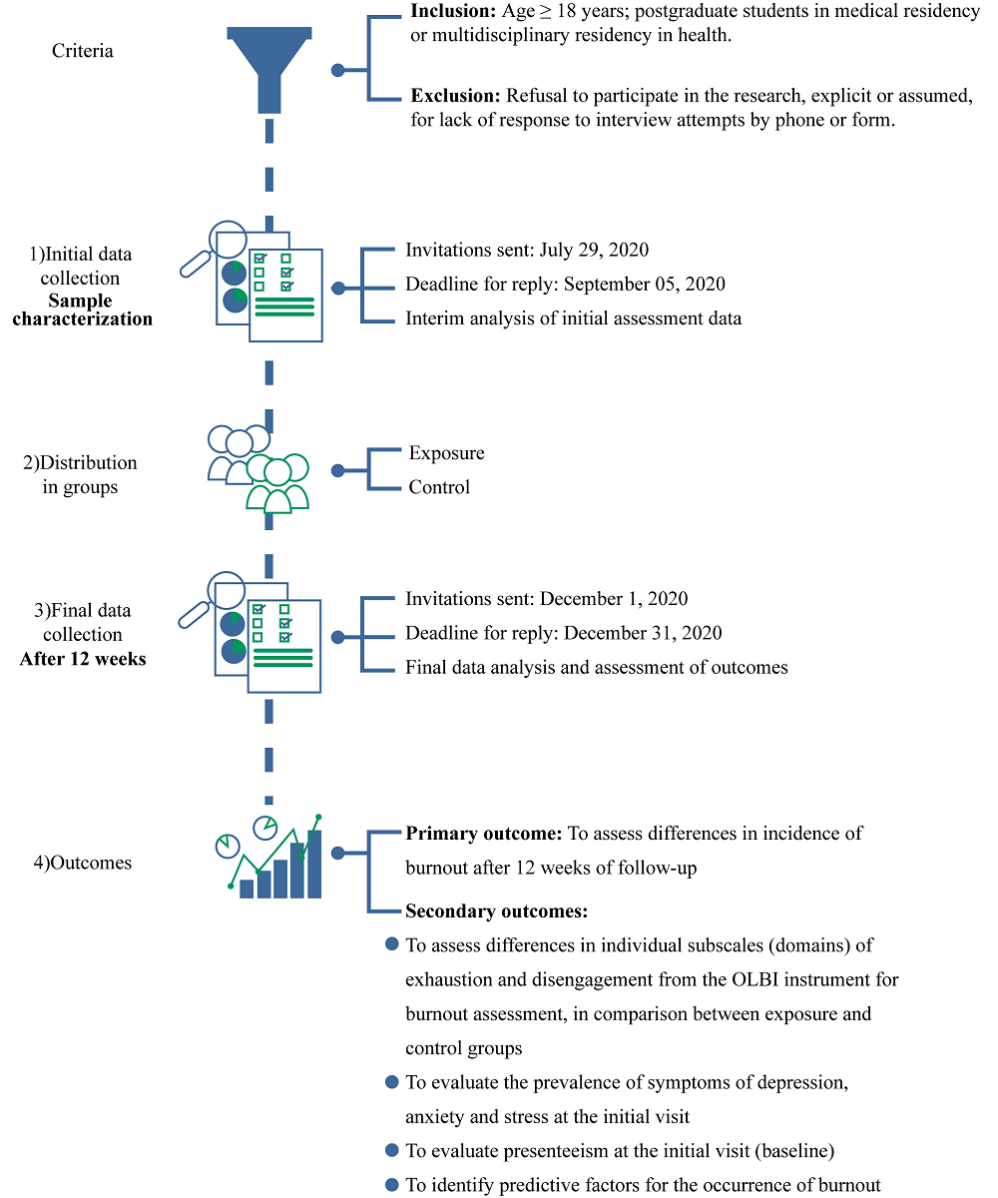
Study fluxogram.

Individuals were recruited from July to August 2020 via electronic invitations sent out through the Microsoft Forms platform (Microsoft Corp). Two waves of data collection were programmed, including initial data collection and a 12-week follow-up (Figure 1). In the first wave (July 29 to September 5, 2020), all data related to the characterization of the study sample, including psychosocial assessment and potential predictive factors related to the research outcomes, were collected. In the second wave of data collection (ie, at the 12-week follow-up), the incidence of burnout will be evaluated, which will then be correlated with the potential predictive factors collected during the first wave. Additionally, we will collect data on participants’ withdrawal from work.

### Research Instruments

#### Depression, Anxiety, and Stress Scale

The Depression, anxiety, and stress scale–21 items (DASS-21) scale has been translated and validated in Portuguese [8] and consists of 3 subscales with 7 items each. Responses are given on a 4-point scale, ranging from 0 (strongly disagree) to 3 (totally agree). The DASS-21 covers 3 symptom domains: depression, anxiety, and stress. The cutoff points for each of these domains are as follows: depression >9, anxiety >7, and stress >14 (Textbox 1, [9]).

**Depression, Anxiety, and Stress Scale–21 items (DASS-21) [9]**

**DASS-21**

**Responses:**

0: Did not apply to me at all

1: Applied to me to some degree, or some of the time

2: Applied to me to a considerable degree or a good part of the time

3: Applied to me very much or most of the time

**Items:**

1. I found it hard to wind down.
2. I was aware of the dryness of my mouth.
3. I couldn’t seem to experience any positive feeling at all.
4. I experienced breathing difficulty (eg, excessively rapid breathing, breathlessness in the absence of physical exertion).
5. I found it difficult to work up the initiative to do things.
6. I tended to overreact to situations.
7. I experienced trembling (eg, in the hands).
8. I felt that I was using a lot of nervous energy.
9. I was worried about situations in which I might panic and make a fool of myself.
10. I felt that I had nothing to look forward to.
11. I found myself getting agitated.
12. I found it difficult to relax.
13. I felt down-hearted and blue.
14. I was intolerant of anything that kept me from getting on with what I was doing.
15. I felt I was close to panic.
16. I was unable to become enthusiastic about anything.
17. I felt I wasn’t worth much as a person.
18. I felt that I was rather touchy.
19. I was aware of the action of my heart in the absence of physical exertion (eg, sense of heart rate increase, heart missing a beat).
20. I felt scared without any good reason.
21. I felt that life was meaningless.

#### Patient Health Questionnaire

Patient Health Questionnaire–9 items (PHQ-9) is a rapid assessment tool that has been translated and validated in Portuguese. It has advantages over other instruments currently validated for use in Brazil [10]. It consists of 9 questions for screening depression, with respondents expected to mark responses in relation to the frequency of symptoms they have experienced in the last 2 weeks: 0, no day; 1, less than 1 week; 2, 1 week or more; and 3, almost every day (Textbox 2, [11]).

**Patient Health Questionnaire–9 items (PHQ-9) [11].**

**PHQ-9**

**Responses:**

0: Not at all

1: Several days

2: More than half the days

3: Nearly every day

**Items:**

1. Little interest or pleasure in doing things
2. Feeling down, depressed, or hopeless
3. Trouble falling or staying asleep, or sleeping too much
4. Feeling tired or having little energy
5. Poor appetite or overeating
6. Feeling bad about yourself—or that you are a failure or have let yourself or your family down
7. Trouble concentrating on things, such as reading the newspaper or watching television
8. Moving or speaking so slowly that other people could have noticed? Or, so fidgety or restless that you have been moving a lot more than usual?
9. Thoughts that you would be better off dead, or thoughts of hurting yourself in some way

#### Brief Resilient Coping Scale

Brief Resilient Coping Scale (BRCS) is a 1D instrument comprising 4 items to assess an individual’s ability to deal with stress in an adaptive way [12]. The responses to the questionnaire items are provided on a 5-point scale: 5, almost always; 4, very often; 3, often; 2, occasionally; and 1, almost never. Total scores on the scale range from 4 and 20, and according to its developers, respondents who score less than 13 are considered to have a low level of resilience (Textbox 3, [13]).

**Brief Resilient Coping Scale (BRCS) [13].**

**BRCS**

**Responses:**

1: Does not describe me at all

2: Does not describe me

3: Neutral

4: Describes me

5: Describes me very well

**Items:**

1. I look for creative ways to alter difficult situations.
2. Regardless of what happens to me, I believe I can control my reaction to it.
3. I believe I can grow in positive ways by dealing with difficult situations.
4. I actively look for ways to replace the losses I encounter in life.

#### Autonomy Degree Scale to Decide Conduct at Work

This visual analog scale (VAS) assesses an individual’s perception of autonomy at their job. Response options range from 0 to 10, with 0 indicating “I have no autonomy” and 10 indicating “I have total autonomy.” Total scores ≤4 indicate a low level of perceived autonomy at work [14].

#### Perception of Availability of Personal Protective Equipment

This single-item instrument was used to assess the availability of personal protective equipment (PPE) for health professionals on a scale of 1 to 5. The question was “In your professional practice, in patient care, for which period of time did you have sufficient and adequate personal protective equipment (PPE) available?” The response options were as follows: 1, no time; 2, less than half the time; 3, half the time; 4, more than half the time; and 5, all the time.

#### Oldenburg Burnout Inventory

The Oldenburg Burnout Inventory (OLBI) is used to assess burnout through the development of a cross-culturally adapted version for both Brazil and Portugal [15]. The OLBI is a 5-point self-reported scale: 5, strongly agree; 4, agree; 3, neither agree nor disagree; 2, disagree; and 1, strongly disagree. This 2D scale covers disengagement and exhaustion, and each dimension comprises 8 items. The disengagement dimension refers to the distancing from work in terms of object and content and the development of cynical and negative attitudes and behaviors toward work. The exhaustion dimension refers to feelings of physical fatigue, need for rest, feeling of overload, and emptiness in relation to work (Textbox 4, [16]).

**Oldenburg Burnout Inventory (OLBI) [16].**

**OLBI**

**Responses:**

1: Strongly disagree

2: Disagree

3: Neutral

4: Agree

5: Strongly agree

**Items:**

**Disengagement**

1. I always find new and interesting aspects in my work.

3. It happens more and more often that I talk about my work in a negative way.

6. Lately, I tend to think less at work and do my job almost mechanically.

9. I find my work to be a positive challenge.

11. Sometimes I feel sickened by my work tasks.

13. This is only type of work that I can imagine myself doing.

15. I feel more and more engaged in my work.

**Exhaustion**

2. There are days when I feel tired before I arrive at work.

4. After work, I tend to need more time than in the past in order to relax and feel better.

5. I can tolerate the pressure of my work very well.

8. During my work, I often feel emotionally drained.

10. After working, I have enough energy for my leisure activities.

12. After my work, I usually feel worn out and weary.

14. Usually, I can manage the amount of my work well.

16. When I work, I usually feel energized.

#### External Work Contract

This instrument comprises a single “yes” or “no” item to assess the existence of an employment relationship rather than that of a residency.

#### Providing Care for Patients With COVID-19

This instrument forms a single “yes” or “no” item to assess whether the respondent provides direct assistance to patients with COVID-19.

#### Stanford Presenteeism Scale

The Stanford Presenteeism Scale (SPS-6) exclusively assesses presenteeism. This instrument helps evaluate the relationship between presenteeism, health problems, and productivity among workers. It consists of 6 items, with responses for each item ranging from 1 (strongly disagree) to 5 (strongly agree) (Textbox 5, [17]).

**Stanford Presenteeism Scale (SPS-6) [17].**

**SPS-6**

**Directions:**

Please describe your work experiences in the past month. These experiences may be affected by many environmental as well as personal factors and may change from time to time. For each of the statements below, please check one of the following responses to indicate your agreement or disagreement with the statement in describing your work experiences in the past month.

**Responses:**

Please use the following scale for evaluation:

1: I strongly disagree with the statement.

2: I somewhat disagree with the statement.

3: I am uncertain about my agreement with the statement.

4: I somewhat agree with the statement.

5: I strongly agree with the statement.

**Items:**

1. Because of my (health problem)*, the stresses of my job were much harder to handle.
2. Despite having my (health, problem)*, I was able to finish hard tasks in my work.
3. My (health problem)* distracted me from taking pleasure in my work.
4. I felt hopeless about finishing certain work tasks, due to my (health problem)*.
5. At work, I was able to focus on achieving my goals despite my (health problem)*.
6. Despite having my (health problem)*, I felt energetic enough to complete all my work.

*Note: the words “back pain,” “cardiovascular problem,” “illness,” “stomach problem,” or other similar descriptors can be substituted for the words “health problem,” in any of these items.

### Participants and Eligibility Criteria

The eligibility criteria used for the inclusion of participants was as follows: aged 18 years or above and postgraduate student in a medical residency or multidisciplinary residency program who has been designated for activities that involve direct patient care during the COVID-19 pandemic. The exclusion criteria have been defined as the explicit or assumed refusal to participate in the study as indicated by no response to telephone or electronic form interview attempts.

### Study Groups

For longitudinal tracking purposes, the exposed groups (parallel to each other) will comprise participants who are farther away from normal or reference ranges, in an unfavorable sense. This will be determined based on the following cutoff points defined for each of the instruments selected (ie, scales to assess burnout, depression, anxiety, stress, and resilience):

- DASS-21 scores
  Depression >9
  Anxiety >7
  Stress >14
- PHQ-9: scores ≥9 indicate depression
- BRCS: scores ≤13 indicate low resilience

In the exposed groups, we will also include participants with lower scores on the evaluation instruments for measuring autonomy at work, availability of PPE, and subjective perceptions of pedagogical adequacy of the residency program, according to the cutoff points listed below. Participants who have a work contract outside the residency program and who are directly involved in care provided to patients with COVID-19 will also be part of the exposed group. The following elements will be considered as predictors of burnout:

- Autonomy degree scale to decide conduct at work (VAS): score ≤4
- Availability and adequacy of PPE for assistance activities (5-point Likert scale): score ≤3
- Proper pedagogical organization of the medical residency program or multidisciplinary (VAS): score ≤5
- External working contract: yes
- Providing care to patients with COVID-19: yes

The corresponding control groups will include participants who present burnout levels considered to be minimally satisfactory or close to the normal or reference values based on the following cutoff points defined for each of the instruments. Participants who do not have a working contract external to the residency program and who are not directly involved in the care provided to patients with COVID-19 will also be part of the control group.

- DASS-21 scores
  Depression ≤9
  Anxiety ≤7
  Stress ≤14
- PHQ-9: score <9
- BRCS: score >13
- Autonomy degree scale to decide conduct at work (VAS): score >4
- Availability and adequacy of PPE for assistance activities (5-point Likert scale): score >3
- Proper pedagogical organization of the medical residency program or multidisciplinary (VAS): score >5
- External working contract: no
- Providing care to patients with COVID-19: no

### Sampling Size

In estimating the required sample size, a general prevalence of about 28% for burnout syndrome among health care professionals was taken as the starting point [18]. Nevertheless, higher burnout prevalence should be expected among individuals with relevant predisposing factors. In order to identify such risk factors, we considered a difference of at least 10 percent points in the prevalence of burnout between the exposed and nonexposed groups as clinically relevant. Therefore, aiming to detect at least a 10-percent-point difference between the groups after 12 weeks of follow-up, the total sample size was initially calculated at N=686 (ie, n=343 for each group). However, only about 72% of the participants initially included (ie. those who did not exhibit burnout at the initial assessment) should enter the longitudinal phase and be analyzed after 12 weeks. Moreover, a dropout rate of up to 20% was expected during this longitudinal follow-up. To compensate for these expected losses, the minimum sample size was thus recalculated to be N=1144 (n=572 in each group). Electronic forms were sent to all participants to collect relevant data for the research, with additional clarifications sought from the participants by telephonic contact, if necessary.

### Clinical Data and Outcomes

The primary outcome will assess the differences in the incidence of burnout (determined using the OLBI instrument) between the exposure and control groups at 12 weeks of follow-up. Participants will be classified as “experiencing burnout” if their exhaustion score is ≥2.25 and their disengagement score is ≥2.10, considering the achieved outcome (clinically relevant difference) in case of a ≥10% (relative risk ≥1.10) difference in the occurrence of burnout between groups.

Secondary outcomes will assess differences in exhaustion and disengagement scores, as determined using the OLBI instrument to assess burnout between the exposure and control groups, with 12 weeks of follow-up. The outcome will be considered if relative risk ≥1.15, resulting in scores of ≥2.25 and ≥2.10 for exhaustion and disengagement, respectively. The prevalence of depression, anxiety, and stress symptoms at the initial visit, as measured by DASS-21 and PHQ-9 and based on previously defined cutoff scores, will also be considered as secondary outcomes. Furthermore, SPS-6 will be administered at the baseline, with a cutoff score for clinical relevance <18, in case of a difference of ≥15% (OR≥1.15) when comparing the exposure and control groups. Absence from work in the previous 12 weeks will be evaluated descriptively through a survey (administered via Microsoft Forms) at the final evaluation stage (Figure 1). Risk factors for the occurrence of burnout will also be evaluated at the 12-week follow-up and compared between the exposure and control groups, as evaluated by relative risks and 95% CIs. An interim analysis with data from the initial assessment (ie, cross-sectional data) will be performed to estimate the prevalence as soon as the recruitment of participants is completed.

### Statistical Analysis

Outcomes based on proportions will be compared between the exposed and control groups by using the chi-square test (or Fisher exact test). Outcomes based on continuous variables will be compared by Student *t* test (or Mann-Whitney U test). The predictive factors for burnout among the candidates will be evaluated using a generalized linear model log-binomial.

For the longitudinal follow-up, participants with scores indicative of burnout at the baseline assessment will be excluded from incidence analyses at the 12-week follow-up. Primary and secondary outcomes will be compared between participants of different professional categories (ie, medical residents and multidisciplinary residents), and potential imbalances observed between groups (ie, exposed vs control groups) will be adjusted by multiple linear regression, logistically or log-binomial, as appropriate.

### Ethics and Dissemination

This study was approved by the Research Ethics Committee from the Medical School (CEP/FM) of the University of Brasília (CAAE: 33493920.0.0000.5558), through the CEP/CONEP system - Plataforma Brasil in 05/07/2020. An informed consent form will be obtained from all participants included in the study. As this is an observational study, one of the biggest risks perceived by the participants is the eventual discomfort in the face of any personal questions that may be part of the initial clinical interview conducted to determine the application of research instruments. The protocol will be registered in the Brazilian Clinical Trials Registry (Registro Brasileiro de Ensaios Clínicos) as an observational study. Undergraduate students in medicine and other undergraduate health courses will participate in the study as collaborating researchers.

## Results

Data collection for this study is currently in progress. Recruitment (for the first phase) started on July 29, 2020, and ended on September 5, 2020. Analyses of data collected during this first phase is already in progress, and we estimate this to be completed by January 2021. The second phase of data collection was lauched on December 1, 2020, and we expect it to be completed by December 31, 2020; thereafter, we will begin analyses of the data collected in this phase.

## Discussion

This prospective cohort study will help evaluate the prevalence of symptoms that are suggestive of mental disorders and burnout syndrome among postgraduate students of medical and multidisciplinary residencies in Brazil, as well as to determine the predictors of burnout during the COVID-19 pandemic. It is known that heath care workers, in general, have encountered worsened mental health and well-being as a result of the COVID-19 pandemic [19]. Moreover, since studies on this topic are quite limited especially with regard to this study population, we believe that our dataset will help to better understand and evaluate the impact of the ongoing pandemic on the mental health of these professionals; this is extremely relevant not only to scale the consequent losses but also to contribute to the planning and implementation of appropriate measures that can potentially alleviate these challenges in the near future.

## Abbreviations

BRCS: Brief Resilient Coping Scale
DASS-21: Depression, anxiety, and stress scale, 21 items
OLBI: Oldenburg Burnout Inventory
PHQ-9: Patient Health Questionnaire, 9 items
PPE: personal protective equipment
SPS-6: Stanford Presenteeism Scale
VAS: visual analog scale
